# The complete mitochondrial genome of *Ricania shantungensis* (Hemiptera: Ricaniidae) in Korea

**DOI:** 10.1080/23802359.2020.1840941

**Published:** 2020-12-24

**Authors:** Jae-Yeon Kang, Injung An, Soyeon Park

**Affiliations:** Evolutionary Ecology Research Team, National Institute of Ecology, Seocheon, Republic of Korea

**Keywords:** *Ricania shantungensis*, Ricaniidae, planthopper, mitochondrial genome

## Abstract

In this study, we have sequenced and annotated the complete mitochondrial genome of *Ricania shantungensis* (Hemiptera: Ricaniidae) for the first time. The circular mitogenome of *R. shantungensis* was 15,789 bp, including 13 protein-coding genes, two ribosomal RNA genes, 22 transfer RNAs, and a single control region of 1,363 bp. Its AT ratio was 74.6%. According to the phylogenetic tree, *R. shantungensis* was clustered with the genus *Ricania*.

It has been reported that there are 40 genus and 400 species in the family Ricaniidae, including *Ricania shantungenesis* and the genus *Ricania* includes about 40 species distributed in Asia including China and India (Xu et al. 2006). The original distribution of *R. shantungensis* is assumed to be Zhejiang and Shandong provinces in China (Chou and Lu 1977). It damages host plants by sucking and spawning and causes sooty mold disease. This incurs economic losses for various fruit trees such as blueberries and apples. Recently, it has been continuously spreading throughout Korea and has been detected in 43 cities (Kim et al. 2015). In this study, we determined the mitogenome of *R. shantungensis*.

In August 2018, the sample was collected from Daejeon, Korea (36.114775 N, 127.330402E). Genomic DNA was extracted using DNeasy Blood & Tissue Kit (QIAGEN, Hilden, Germany). The specimen’s genome DNA (Accession number: 973) was deposited in the National Institute of Ecology, Seocheon, Korea. Raw sequences obtained from Illumina HiSeqX (Macrogen Inc., South Korea) were filtered by Trimmomatic 0.33 (Bolger et al. 2014) and *de novo* assembled by Velvet 1.2.10 (Zerbino and Birney 2008) and gap sequences were closed with SOAPGapCloser 1.12 (Zhao et al. 2011), BWA 0.7.17, and SAMtools 1.9 (Li et al. 2009; Li 2013). Geneious R11 11.1.5 (Biomatters Ltd, Auckland, New Zealand) was used to annotate the mitochondrial genome of *R. shantungensis* with referring mitogenomes of *R. speculum* (NC_031369 and MT834932) and *R. marginalis* (NC_019597).

The complete mitogenome of *R. shantungensis* (GenBank accession number: MT898421) was 15,789 bp with 74.6% AT content, which was longer than any other available *Ricania* mitochondrial genome (*R. speculum*: 15,729bp). It contained all 39 genes of a standard insect mitochondrial genome, including 13 protein-coding genes, two rRNAs, and 22 tRNAs. The order of the 39 genes was conserved as in all other hemipteran mitogenomes. The single large non-coding control region was also found in the mitogenome, and was 1,363 bp long, the largest of all known *Ricania* mitogenomes.

We inferred phylogenetic relationships based on multiple sequence alignments of 16 planthopper mitogenomes including two outgroup species, *Magicicada tredecula* (NC_041656) and *Philaenus spumarius* (NC_005944) (Song and Liang 2013) except variable control regions. Multiple sequence alignment was conducted using MAFFT 7.450 (Katoh and Standley 2013). Phylogenetic trees were constructed by maximum likelihood (ML) using MEGA X (Kumar et al. 2018) and Bayesian inference (BI) using MrBayes 3.2.6 (Ronquist et al. 2012). The tree topology of ML and BI were identical (Figure 1). According to the tree, *R. shantungensis* was clustered with two species, *R. marginalis* and *R. speculum*, belonging to the genus *Ricania*. In addition, Ricaniidae was included in a clade with Flatidae. This study will be helpful to understand the phylogenetic relationships of Ricaniidae.

**Figure 1.**
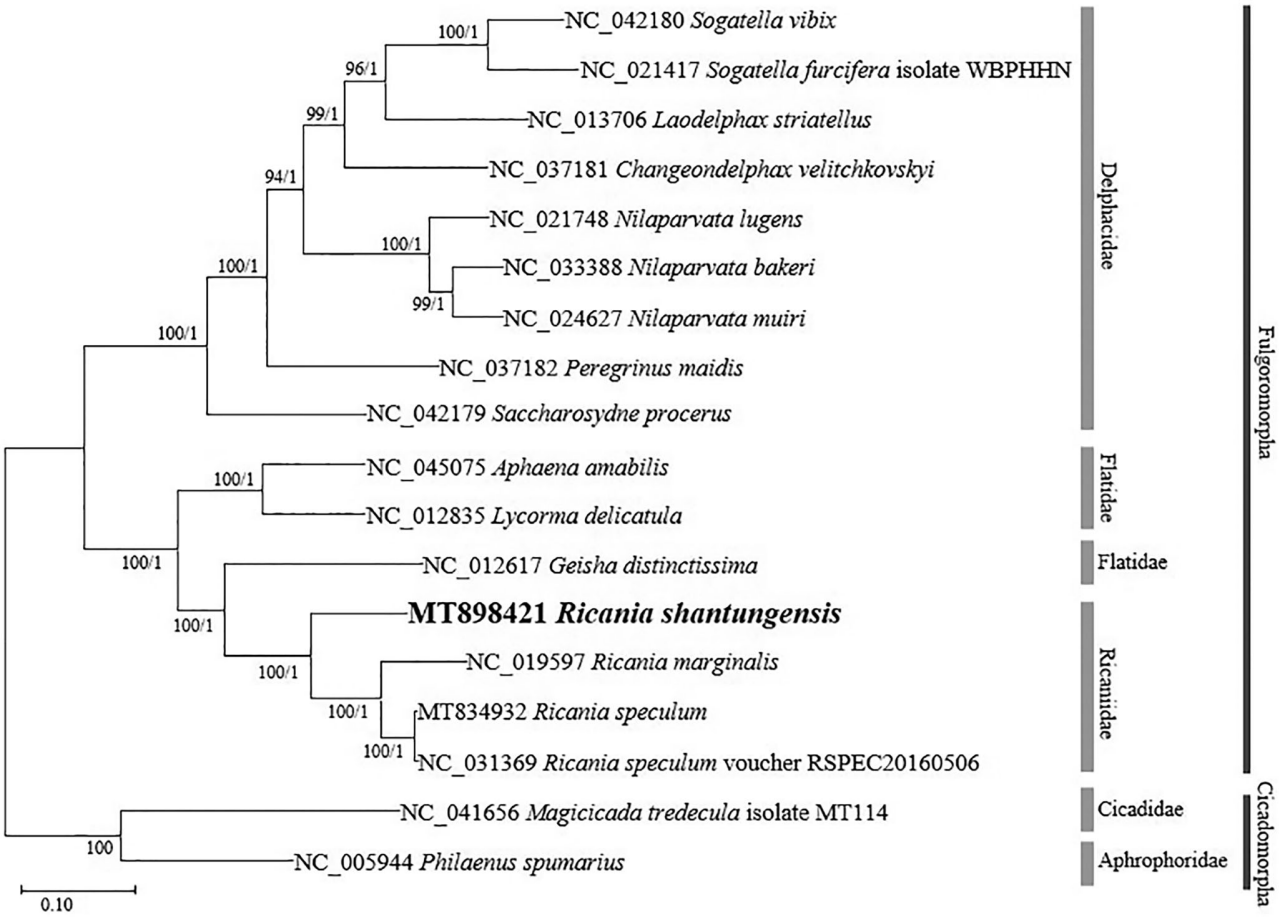
Maximum likelihood (1,000 bootstrap repeats) and Bayesian inference phylogenetic trees of 16 Fulgoromorpha mitochondrial genomes including two outgroup species (*Magicicada tredecula* and *Philaenus spumarius*). Numbers on internodes indicate maximum likelihood bootstrap poportions (left) and Bayesian posterior probabilities (right).

## Data Availability

The data that support the findings of this study are openly available in GenBank at https://www.ncbi.nlm.nih.gov/genbank/, accession number [MT898421].
